# Efficacy of the Suboccipital Paracondylar-Lateral Cervical Approach: The Series of 64 Jugular Foramen Tumors Along With Follow-Up Data

**DOI:** 10.3389/fonc.2021.660487

**Published:** 2021-10-14

**Authors:** Xiangyu Wang, Jian Yuan, Dingyang Liu, Yuanyang Xie, Ming Wu, Qun Xiao, Chaoying Qin, Jun Su, Yu Zeng, Qing Liu

**Affiliations:** Department of Neurosurgery in Xiangya Hospital, Central South University, Changsha, China

**Keywords:** schwannomas, jugular foramen, skull base neoplasms, surgical approach, meningiomas

## Abstract

**Objective:**

Complete resection of jugular foramen tumors with minimal cranial nerve complications remains challenging even for skilled neurosurgeons. Here, we introduce a modified paracondylar approach, named the suboccipital paracondylar-lateral cervical (SPCLC) approach for this purpose. We also share the follow-up data of our series and discuss the advantages and limitations of this modified paracondylar approach.

**Methods:**

We included 64 patients with jugular foramen tumors who underwent surgery by the same senior neurosurgeon between November 2011 and August 2020. All patients were treated with the SPCLC approach, which aimed for gross total tumor removal in a single-stage operation. The clinical characteristics, including preoperative and postoperative neurological status, the extent of surgical resection, and follow-up data were retrospectively acquired and evaluated.

**Results:**

There were 48 schwannomas, nine meningiomas, three paragangliomas, one hemangiopericytoma, one chordoma, one endolymphatic sac tumor, and one Langerhans’ cell histiocytosis. The median age of our patients was 43 years (range: 21–77 years). Dysphagia, hoarseness, and tongue deviation were observed in 36, 26, and 28 patients, respectively. Thirty-two patients had hearing function impairments, including hearing loss or tinnitus. Gross total resection was achieved in 59 patients (59/64, 92.2%). Gamma Knife treatment was used to manage residual tumors in five patients. Postoperatively, new-onset or aggravative dysphagia and hoarseness occurred in 26 and 18 cases, respectively. Nine patients developed new-onset facial palsy, and one patient developed new-onset hearing loss. There were no cases of intracranial hematoma, re-operation, tracheostomy, or death. At the latest follow-up, hearing loss and tinnitus had improved in 20 cases (20/32, 62.5%), dysphagia alleviated in 20 cases (20/36, 55.6%), and hoarseness improved in 14 cases (14/26, 53.8%). Over a mean follow-up period of 27.8 ± 19.5 months (range: 3–68 months), tumor recurrence was observed in one patient.

**Conclusion:**

The SPCLC approach, modified from the paracondylar approach, and was less invasive, safe, and efficient for certain jugular foramen tumors. Taking advantage of the anatomic understanding, clear operational vision, and appropriate surgical skills, it is possible to achieve gross total tumor removal and the preservation of neurological function.

## Introduction

Jugular foramen (JF) tumors can extend into the intracranial and parapharyngeal spaces through the jugular foramen. Common JF tumors include paragangliomas, schwannomas, and meningiomas (1, 2). Due to the anatomical complexity of this area, surgery usually places the lower cranial nerve (CN) and vertebral and cerebellar arteries at high risk. Most JF tumors in this study were JF schwannomas (JFSs); therefore, this study focuses on JFS treatment. According to the classification proposed by Samii et al., JFSs can be divided into four types (3, 4). The retrosigmoid approach is an intradural approach leading to the JF *via* its medial side and is best indicated for lesions located in the posterior fossa, such as type A JFSs (3, 5–7). However, the removal of type B, C, and D JFS tumors remain challenging. In particular, for dumbbell-like JF tumors, a combined approach or multi-disciplinary cooperation is required.

Since the pathological characteristics and growth patterns are different between different tumors, the surgical approach should be selected individually. The growth of JFSs is non-infiltrative; the extent of most is often limited in the post-styloid space. In contrast to paragangliomas and meningiomas, bone infiltration and internal carotid artery (ICA) encasement are seldom observed in patients with JFSs. We used a modified paracondylar approach, named the suboccipital paracondylar-lateral cervical (SPCLC) approach, which was suitable for the removal of most JFSs and well-selected small jugular foramen meningiomas and paragangliomas.

## Methods

**Figure 1:**
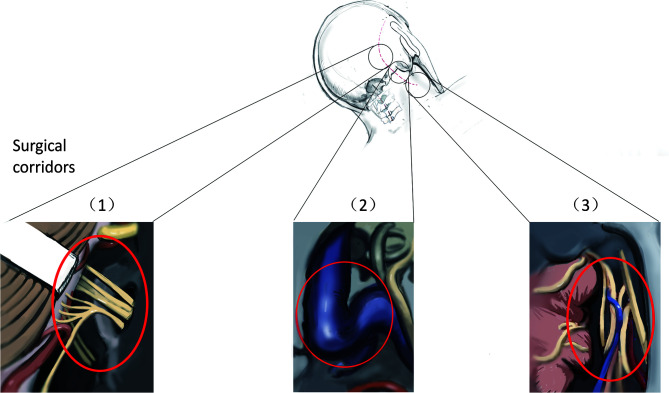
Three surgical corridors of suboccipital paracondylar-lateral cervical approach. (1) The suboccipital retrosigmoid corridor exposes the cerebellopontine angle cistern and cerebellomedullary angle cistern; (2) The paracondylar corridor exposes the jugular foramen; (3) The lateral cervical corridor exposes the retrostyloid region of parapharyngeal space.

This study included 64 patients with JF tumors. All patients underwent single-stage surgery performed by the same neurosurgeon between November 2011 and August 2020. Before the operation, high-resolution computed tomography (HRCT) and contrast-enhanced magnetic resonance imaging (MRI) of the skull base and parapharyngeal space were performed. The goal of the operation was gross total tumor removal while preserving neurological function. The SPCLC approach comprises three different surgical corridors: the suboccipital retrosigmoid, paracondylar, and retrostyloid spaces (**Figure 1**). Based on the anatomical spaces occupied by the tumor, the surgeon can select the appropriate surgical corridors needed from the SPCLC approach (**Table 1**).

**Table 1:** Tumor classifications and surgical details.

Tumor Type	Tumor occupied spaces	No. of cases	No. of GTR cases	Surgical corridors	Additional bone removal
Schwannomas	Cistern	9	9	(1)	
	JF + Cistern	9	9	(1)+(2)	
	JF	5	5	(2)	Condyle (hypoglossal)
	JF + Retrostyloid	5	5	(2)+(3)	
	Dumbbell-like	20	17	(1)+ (2)+(3)	
Meningioomas	Cistern	3	2	(1)+ (2)	
	JF + Cistern	2	2	(1)+ (2)	
	Dumbbell-like	4	3	(1)+ (2)+(3)	
Paragangliomas	JF + Retrostyloid	2	2	(1)+ (2)	Infra-labyrinthine
	Dumbell-like	1	1	(1)+ (2)+(3)	
Hemangiopericytomas	JF+Retrostyloid space	1	1	(2)+(3)	
Chordoma	JF+Cistern	1	1	(1)+ (2)	
Endolymphatic sac tumor	JF+labyrinth	1	1	(2)	Infra-labyrinthine and post-labyrinthine
Langerhans’cell histiocytosis	Dumbell-like	1	1	(1)+ (2)+(3)	

### Position and Skin Incision

**Figure 2:**
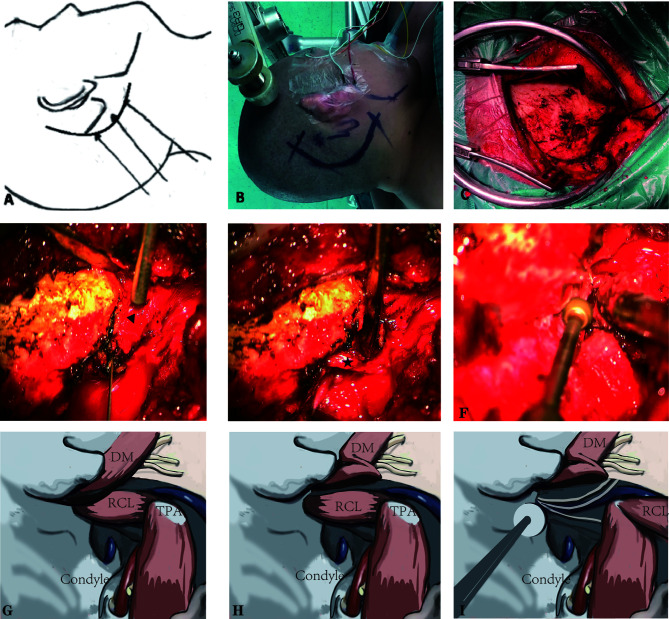
The suboccipital paracondylar-lateral cervical (SPCLC) approach. **(A)** Schematic diagram of the SPCLC approach, the approach accesses the dumbbell-like jugular foramen schwannomas by three compartments: the posterior fossa, jugular foramen, and retrostyloid region; **(B)** The head position and the incision; **(C)** The cutaneous muscle flap elevated to expose the suboccipital area and retrostyloid region; **(D, E)** The post belly of the digastric muscle (triangle) and rectus capitis lateralis (asterisk) identified; **(F)**, The jugular process was removed. **(G–I)** The schematic diagram of **(D–F)**. DM, digastric muscle; RCL, rectus capitis lateralis; TPA, transverse process of the atlas.

The patients were placed in the lateral prone position with the head rotated away from the side of the lesion to reveal the highest point of the mastoid. Using the SPCLC approach, the atlanto-occipital joint was extended (**Figures 2A, B**). Thus, the space between the atlas and ramus of the mandible was extended to facilitate the exposure and resection of tumors in the poststyloid area of the parapharyngeal space. A retro-auricular, inverted L-shaped incision or C-shaped incision was then used (**Figures 2A, B**). We preferred the C-shaped incision because it was easier to retract the cutaneous muscle flap anteriorly and expose the surgical area. Based on the lower margin of the tumor, the lower margin of the incision can be extended to the level of the mandibular angle. If the intradural tumor maximal diameter is larger than 4 cm, the L-shaped incision should be utilized since it may provide a bigger suboccipital window for tumor removal. The upper margin of the incision was the superior nuchal line which indicated that the location of the transverse sinus and the right-angle corner was located at the asterion, which was used to define the transition between the transverse and sigmoid sinuses. The orbicularis oculi muscle, the orbicularis oris muscle, and the mentalis were monitored by Medtronic NIM-Eclipse E4 software through Subdermal Needle electrodes (SD51-426-1 NeuroDart, Spes Medica, Italy; 0.4mm diameter, 13mm length, with 1.5mm touch-proof connector and 100cm cable length).

### Craniotomy

Firstly, the cutaneous muscle flap was retracted from the occipital bone (**Figure 2C**). Secondly, the post belly of the digastric muscle was determined in the digastric muscle groove, which is posterior to the mastoid tip (**Figures 2D, G**). This muscle is the first critical landmark of the approach since its muscle fascia goes anteriorly and adjacent to the stylomastoid foramen. Taking advantage of the neuro-electrophysiological monitoring, it would not be difficult to locate the facial nerve that exits the stylomastoid foramen. Such a procedure may be helpful for the precaution of facial nerve injury. Moreover, the post belly of the digastric muscle serves as the anterosuperior boundary of the Henry fat gap, which provides the natural corridor to the retrostyloid space posteriorly. The most important portion of craniotomy is locating and drilling the jugular process. To located the jugular process, the transverse process of the atlas (TPA) and the rectus capitis lateralis (RCL) are the key landmarks. The TPA bisects the line connecting the mastoid tip and the mandibular angle; the surgeons may use their fingers to palpate and locate the TPA. After locating the TPA, the surgeon may easily identify the RCL, which arises from the upper surface of the TPA and attaches to the occipital jugular process (**Figures 2E, H**). Following the previous steps, the jugular process can be precisely identified and removed (**Figures 2F, I**). Finally, the separated posterior fossa bone flap is removed based on the intracranial tumor size. In detail, the anterior border of the bone flap is the posterior margin of the sigmoid sinus while the upper border is below the transverse sinus.

**Figure 3:**
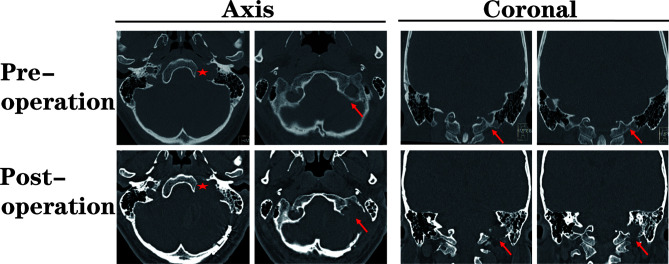
The pre- and post-operational high-resolution computed tomography (HRCT) indicated the extent of paracondylar bone removal. During the suboccipital paracondylar-lateral cervical approach, the jugular process was precisely removed while the condyle was kept intact. Red star: enlarged jugular foramen; Red arrow: the extent of removed bone mass.

Compared with the standard far-lateral approach, there was no need to expose or translocate the vertebral artery in this method. Furthermore, we did not routinely remove the occipital condyle in our patients (**Figure 3**), except when removing the hypoglossal schwannomas originating from the hypoglossal canal.

### Skull Base Reconstruction

**Figure 4:**
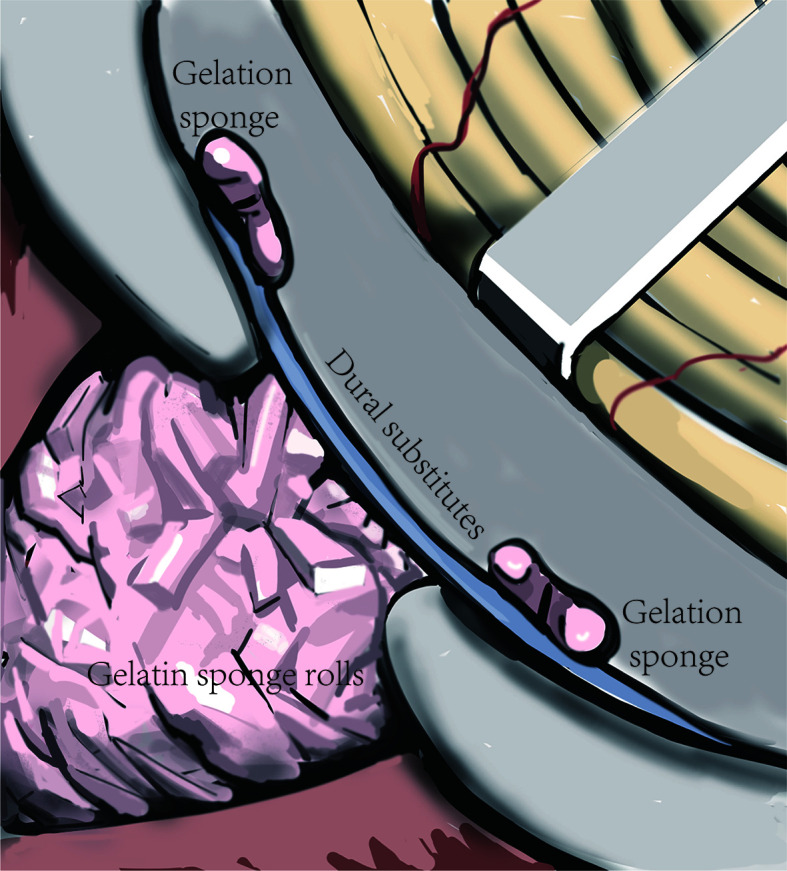
Schematic diagram of the “sandwich” skull base reconstruction method. The dural substitute was placed on the inner face of the skull base. The gelatin sponge rolls were filled up in the tumor cavity to sustain the dural substitute and one layer of gelatin sponge was cover the margin of the dural substitute to keep it in place.

After removing the tumor, we performed skull base reconstruction. Since the dumbbell-like JFSs were located across the JF, defects of the dura and bone around the jugular foramen were inevitable. Using the SPCLC approach, only the paracondylar bone and dura are have defected after surgery. We utilize the “sandwich”‘ method to reconstruct the skull base (**Figure 4**). Initially, the parapharyngeal space was filled up with gelatin sponge rolls. Then, the dural substitute was placed in the inner face of the skull base. Lastly, a single layer of absorbable gelatin sponge was placed around the margin of the dural substitute to keep it in place. After the incision is sutured, the surgical area should be wrapped with pressure to sustain the “sandwich” firmly.

### Postoperative Adjuvant Radiotherapy and Follow-Up

We evaluated the extent of tumor resection based on intraoperative observations and confirmed them *via* postoperative MRI. Gross total resection (GTR) was defined as having no residual tumor observed on postoperative MRI. Subtotal resection was defined as MRI evidence of a residual tumor with a volume of less than 10% of the original tumor size. Patients who underwent subtotal resection underwent Gamma Knife treatment in the third month after surgery to reduce the risk of residual tumor progression. The Perfexion Model (Elekta Instruments, Inc., Stockholm, Sweden) was adopted. The Leksell stereotactic head frame (model G) was positioned and magnetic resonance images (MRI; 1.5-tesla; Magnetom Vision, Siemens, Munich, Germany) were performed. We treated the tumor volume with a marginal dose of 13 Gy at 50% isodose. All patients underwent brain MRI within the first 3 days after surgery. For follow-up, all patients underwent brain MRI at 3, 6, and 12 months after surgery, following which tumor recurrence/progression was assessed each year if possible. In most patients, neurological function was assessed at outpatient follow-up visits, while some patients only engaged in telephone interviews. We retrospectively reviewed the clinical characteristics, the extent of surgical resection, and follow-up data of each patient.

## Results

### Clinical Characteristics

**Figure 5:**
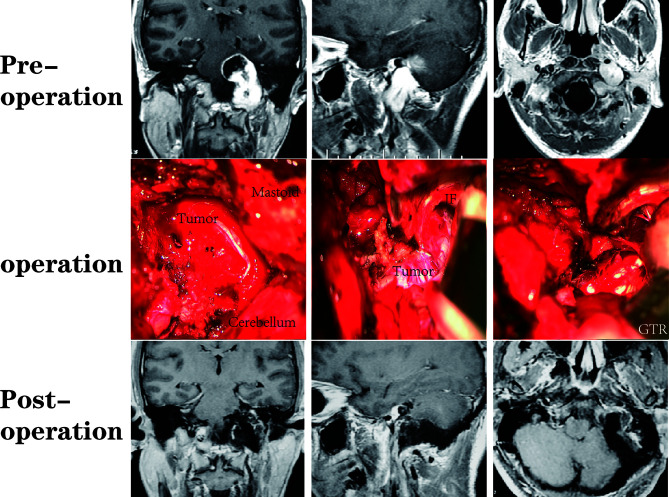
A patient with a JFS who underwent suboccipital paracondylar-lateral cervical approach. The jugular foramen was opened posteriorly by removing the jugular process. The parapharyngeal tumor was removed first, followed by the foramen and the intracranial tumor.

**Figure 6:**
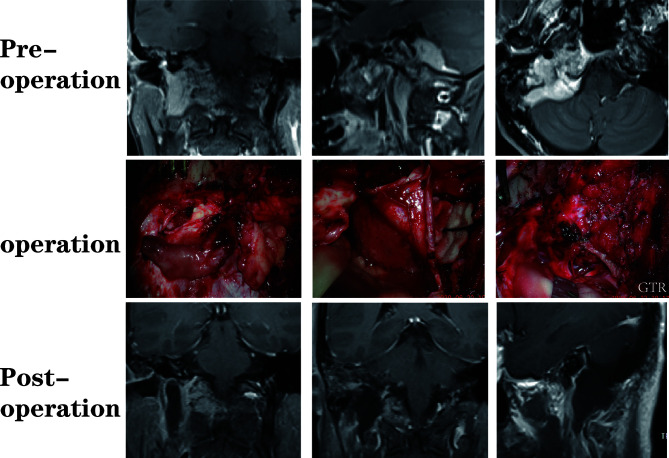
A patient with a dumbbell-like jugular foramen meningioma who underwent suboccipital paracondylar-lateral cervical approach.

**Figure 7:**
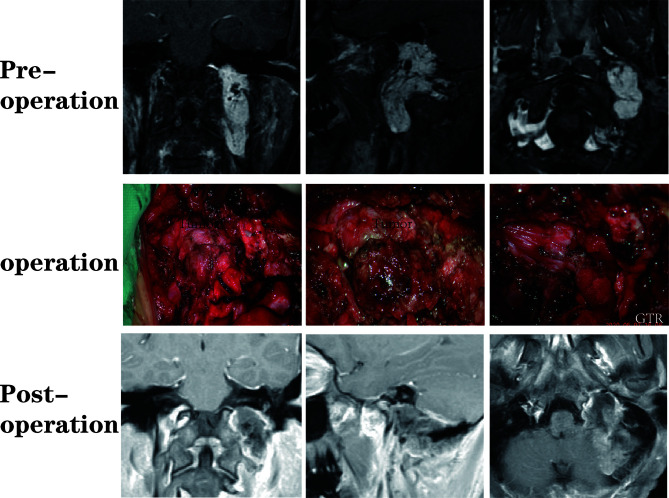
A patient with a jugular foramen paraganglioma who underwent suboccipital paracondylar-lateral cervical approach.

A total of 64 patients (31 male, 33 female) who underwent surgical treatment for dumbbell-like JFSs were enrolled in this study, including 58 cases of primary origin and six cases of recurrent origin. Specifically, there were 48 schwannomas (**Figure 5**), nine meningiomas (**Figure 6**), three paragangliomas (**Figure 7**), one hemangiopericytoma, one chordoma, one endolymphatic sac tumor, and one Langerhans’ cell histiocytosis. The median age at the time of operation was 42 years (range: 21–77 years), and the average age was 44.4 ± 14.6 years. Tumor classifications and surgical details are presented in **Table 1**. At the time of the first diagnosis, the most common cranial nerve deficiencies were dysphagia (n=36) and hearing dysfunction (n=32), followed by tongue deviation (n=28), hoarseness (n=26), and facial paralysis (n=9).

### Surgical Outcomes

GTR was achieved in 59 cases (59/64, 92.2%), while subtotal resection (STR) was achieved in five cases. For meningioma, the removal grade was Simpson II (8). There were no cases of intracranial hematoma, re-operation, tracheostomy, or death. **Table 2** lists the neurological deficits before and after the operation. In this series, 13 patients developed new-onset dysphagia, while 13 experienced worsening dysphagia. Another 13 patients had new-onset hoarseness, five experienced worsening of hoarseness after surgery, nine patients developed new facial palsy, and one patient developed new hearing dysfunction. During the perioperative period, 15 patients were fed through a gastric tube for 7 to 22 days (average of 12.1 ± 4.3 days). Nine patients developed meningitis, while eight developed pulmonary infections. Subcutaneous hydrops was also documented in seven cases.

**Table 2:** Neurological deficits before and after surgery (64 cases).

Complication	Pre-operation	Post-operation		
		Aggravation	New-onset	Total
Hearing dysfunction	32	0	1	1 (1.6%)
Dysphagia	36	13	13	26 (40.6%)
Hoarseness	26	5	13	18 (28.1%)
Tongue deviation	28	2	5	7 (10.9%)
Facial paralysis	9	1	9	10 (15.6%)

### Follow-up

The mean follow-up period in our series lasted for 27.8 ± 19.5 months (range: 3–68 months). One tumor recurrence was observed in the GTR group at 36 months after the operation. For the recurrent and STR cases who received Gamma Knife treatment. The mean follow-up time after the Gamma Knife treatment is 25 ± 20.0 months (range: 9-57). There was no tumor progression at the latest follow-up.

A significant proportion of patients had improved neurological function during follow-up (**Table 3**). Hearing dysfunction (i.e., hearing loss, tinnitus) was the most common symptom (20/32, 62.5%). Improvements in dysphagia were observed in 20 cases (20/36, 55.6%) which may be due to compensation from the contralateral function. Hoarseness improved in 14 (14/26, 52.8%) cases. Improvements in tongue deflection were observed in 14 (14/28, 50%) cases. Alleviation of facial palsy was observed in five (5/9, 55.6%) cases.

**Table 3:** Postoperative improvements in neurological function during follow-up.

Neurological dysfunction	Patients exhibiting improvement	Patients with preoperative deficits	Improvement rate
Hearing dysfunction	20	32	62.5%
Dysphagia	20	36	55.6%
Hoarseness	14	26	52.8%
Tongue deviation	14	28	50%
Facial paralysis	5	9	55.6%

## Discussion

Neurological function, especially the CN function, is critical for the quality of life of patients. It is difficult to completely remove the JF tumors and protect the neurological function due to the tumor involvement of both the intracranial and the parapharyngeal space. Considering that re-operation may increase the incidence of neurological deficiency due to scar formation from the earlier operation (4, 9), most scholars have suggested that surgeons should manage to remove JF tumors in single-stage microsurgery (3, 4, 6, 9–11). Besides, one multicenter study revealed that stereotactic radiosurgery improves risk profiles in patients with residual or newly diagnosed small-volume JFSs (12). Optimal treatment should decrease complications and improve the quality of life of patients. Thus, both microsurgery or stereotactic radiosurgery should be considered based on the specific situation, such as tumor volume, age, and surgical skills. In this study, we utilized the SPCLC approach to achieve gross total removal of JFSs and well-selected small paragangliomas and meningiomas in a single-stage surgical operation. The SPCLC approach is modified from the paracondylar approach and is easier for neurosurgeons to perform. Through the SPCLC approach, the JF tumors can be accessed from three corridors in the single-stage operation as follows: using the suboccipital retrosigmoid corridor exposes the cerebellopontine angle cistern and cerebellomedullary angle cistern; drilling the paracondylar bone, especially removing the jugular process, enables access to the JF; and lateral cervical dissection through the Henry fat gap enables access to the poststyloid region of the parapharyngeal space (**Figure 1**).

### Surgical Corridors Selection

Approach selection should facilitate tumor resection, reduce the risk of CN injury, and minimize approach-related complications. Differing from paragangliomas and meningiomas, JFSs rarely invade the internal jugular vein (IJV) or encase the ICA, and may cross the JF and grow into the posterior fossa and parapharyngeal space. Therefore, the preferred surgical approach for JFSs has progressed from extensive exposure of skull base structures to the more precise removal of the affected structures and minimal vessel exposure. To elucidate the indications and limits of the SPCLC approach, we discussed the operational corridors of the SPCLC approach and compare it with other classic approaches.

For intracranial tumors, otolaryngologists prefer the petro-occipital trans-sigmoid (POTS) approach or the infralabyrinthine approach for JFS removal with minimal brain manipulation (13). In contrast, neurosurgeons prefer to use a classical retro-sigmoid (RS) approach that does not ligate the sigmoid sinus and IJV (3, 5–7). Similar to the RS approach, the SPCLC approach exploits a suboccipital retrosigmoid corridor to access the intracranial part of dumbbell-like JFSs.

The JF can be accessed from three directions: lateral, posterior, and anterior (14, 15). The commonly used lateral approaches are the infralabyrinthine approach and the POTS approach. The infralabyrinthine approach is widely used for the resection of intra-foramen tumors (4, 6). Using the endoscope, Samii et al. (3, 10) utilized the infralabyrinthine approach to remove intra-foramen tumors by drilling the supra-jugular bone. The major corridors are anterior to the sigmoid sinus (SS) in both the infralabyrinthine approach and the POTS approach. Both the infralabyrinthine approach and the POTS approach, in which the major surgical corridors are anterior to the sigmoid sinus (SS). The commonly used posterior approaches are the far-lateral approach and its transcondylar, supracondylar, and paracondylar modifications, in which the major surgical corridors are posterior to the SS (5, 16, 17). In the classical far-lateral approach, there is a risk of vertebral artery injury due to exposure or translocation of the vessel (16). The cadaveric study indicated that removing the jugular process, the major procedure in the paracondylar modification, is the key to expose the posterior gate of the JF (18, 19). These anatomic findings are consistent with our long-time clinical applications, which emphasize that the precise location and removal of the jugular process is key to opening the JF posteriorly. As introduced previously, the SPCLC approach is one of the paracondylar modifications of the far-lateral approach, and the TPA and RCL are the clear landmarks of safety located in the jugular process (**Figure 2**). Unlike the classic or transcondylar far-lateral approach, part of the condyle is not routinely removed, except for some hypoglossal schwannomas. In addition, there is no need to expose or translocate the vertebral artery, and all the processes are lateral to the suboccipital triangle.

The two most common approaches to remove parapharyngeal space tumors are the Fisch A infratemporal approach and the transcervical approach. The parapharyngeal space is divided into two regions by the styloid diaphragm: the prestyloid and retrostyloid regions (20). The retrostyloid region contains major vascular structures (ICA, IJV, etc.) and the extracranial portion of the lower CNs from IX to XII (20). Most JFSs are within the retrostyloid region since the tumors originate from the lower CNs. Using the SPCLC approach enables neurosurgeons to easily and directly access the retrostyloid region *via* the Henry fat gap, which will naturally be expanded due to tumor growth, and will not abscess in the styloid diaphragm. In addition, the facial nerve is not routinely exposed or translocated. If the JF tumors especially for the meningiomas and paragangliomas invade the skull base, the infralabyrinthine, transcervical, and the Fisch A infratemporal approaches should be combined. The Fisch A infratemporal approach is essential when tumors widely involve the infratemporal fossa (21). When the tumors encase the ICA or IJV, the transcervical approach should be combined to control such vital vessels during the operation.

### Neurological Function Preservation

After the operation, new-onset hearing loss was only observed in one patient, and only 10 patients experienced mild facial palsy. In addition, the follow-up data showed that facial palsy, hearing dysfunction, dysphagia, and hoarseness were relieved in half of the patients. This may be due to both the protection of neurological function and contralateral compensation. Our experiences with neurological function preservation are as below.

First, both anatomical knowledge and neuro-electrophysiological monitoring played important roles in neurological function preservation. For example, since the posterior belly of the digastric muscle goes anteriorly in the digastric groove connecting to the stylomastoid foramen just posterior to the styloid process, the posterior belly of the digastric muscle can serve as a landmark to identify and protect the facial nerve during craniotomy. Taking advantage of the neuro-electrophysiological monitoring system and the landmark, the surgeon can detect and locate the facial nerve around the stylomastoid foramen. Moreover, the neuro-electrophysiological system can also detect the nerve course during tumor resection.

Furthermore, a clear operational vision is a guarantee for safe and quick operation, and thus, decreasing tumor blood supply becomes vital. Though the sequence of tumor removal remains a controversial issue for dumbbell-like JFSs, it consists of three parts. First, we preferred to remove the tumors in the retrostyloid region. Subsequently, we removed the tumors in the JF. Finally, we opened the dura of the posterior cranial fossa and removed the tumor in the postcranial fossa. After the tumors located in the parapharyngeal and the JF were removed, we discovered that the blood supply of the tumors was sustainably reduced. Taking advantage of the lower blood supply, the operational vision will be clearer and will facilitate neurological function preservation. The adhesions between the tumor and critical structures such as the brainstem and adjacent nerves and vessels will be released naturally after the posterior fossa tumor is decompressed uniformly and sufficiently. The clear operational field and sufficient tumor decompression will facilitate the resection along with the arachnoid interface; therefore, the surgeon can minimize neurovascular damage.

**Figure 8:**
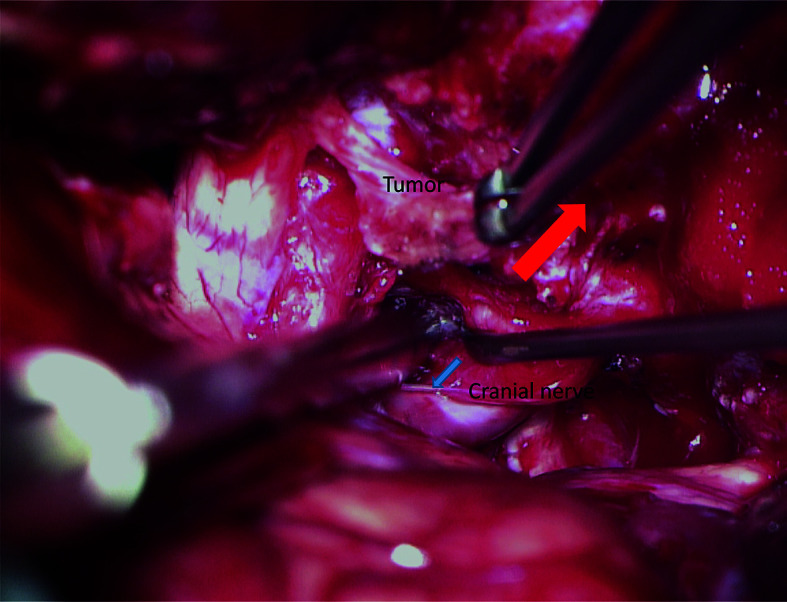
The “*in situ*” technique for the preservation of neurological function. The supporter stably pulls the tumor by the cup forceps to create tension between the tumor and target nerve (red arrow). Using a dissector, the chief surgeon gently divides the tumor away from the nerve along with the arachnoid interface (blue arrow).

Considering that the adjacent nerves are stretched by tumor enlargement, surgeons should separate the tumors from the nerve, rather than pull the nerve away from the tumor (**Figure 8**). In other words, the nerve should stay *in situ* during the separation process. Thus, it is required that the supporter should gently pull the tumor away from the nerve using the cup forceps. This process will create tension between the tumor and the adjacent nerve. Then, the chief surgeon can use the dissector to gently separate the tumor away from the nerve along with the arachnoid interface. According to our experience, this technique will decrease the impairment of neurological function.

### Limitations

The JF meningiomas and paragangliomas in this study were well-selected cases. If the tumors evade the skull base anteriorly or encase the IJV and ICA, multidisciplinary approaches were required to expose more skull base critical structures. In addition, some neurological functions were evaluated mainly based on subjective symptoms, such as hearing recovery and alleviation of dysphagia, which may lead to some bias.

### Conclusion

Since the SPCLC approach precisely opens the JF by removing the jugular process, and directly accesses the retrostyloid region through the Henry fat gap, it provides an alternative for neurosurgeons to safely and efficiently remove the JFSs and other well-selected JF tumors, which did not evade the skull base anteriorly or encase the IJV and ICA. By taking advantage of anatomical understanding, clear operational vision, and appropriate surgical skills, it is possible to achieve gross total tumor removal and the preservation of neurological function.

## Data Availability Statement

The original contributions presented in the study are included in the article/supplementary material. Further inquiries can be directed to the corresponding author.

## Author Contributions

Conception and design: XW and QL. Do the surgery procedure: all authors. Do the Gamma Knife procedure: YZ. Acquisition of data: XW and JY. Analysis and interpretation of data: XW and QX. Operational photo: CQ. Drafting the article: XW. Critically revising the article: all authors. Reviewed submitted version of manuscript: all authors. Approved the final version of the manuscript on behalf of all authors: XW. Study supervision: QL. All authors contributed to the article and approved the submitted version.

## Funding

National Key Technology Research and Development Program of the Ministry of Science and Technology of China (grant number 2014BAI04B01).

## Conflict of Interest

The authors declare that the research was conducted in the absence of any commercial or financial relationships that could be construed as a potential conflict of interest.

## Publisher’s Note

All claims expressed in this article are solely those of the authors and do not necessarily represent those of their affiliated organizations, or those of the publisher, the editors and the reviewers. Any product that may be evaluated in this article, or claim that may be made by its manufacturer, is not guaranteed or endorsed by the publisher.

## Abbreviation

CN, Cranial nerve; GTR, Gross total resection; HRCT, Highresolution computed tomography; ICA, Internal carotid artery; IJV, Internal jugular vein; JF, Jugular foramen; JFS, JF schwannomas; MRI, Magnetic resonance imaging; POTS, Petro-occipital trans-sigmoid; RCL, Rectus capitis lateralis; RS, Retro-sigmoid; SPCLC, Suboccipital paracondylar-lateral cervical; STR, Subtotal resection; TPA, Transverse process of the atlas;VA, Vertebral artery.
